# Modelling and Classification of Apple Textural Attributes Using Sensory, Instrumental and Compositional Analyses

**DOI:** 10.3390/foods10020384

**Published:** 2021-02-10

**Authors:** Masoumeh Bejaei, Kareen Stanich, Margaret A. Cliff

**Affiliations:** 1 Summerland Research and Development Centre, Science and Technology Branch, Agriculture and Agri-Food Canada, Summerland, BC V0H 1Z0, Canada; Kareen.Stanich@Canada.ca; 2 Food Nutrition and Health, Faculty of Land and Food Systems, The University of British Columbia, Vancouver, BC V6T 1Z4, Canada; Margaret.Cliff@ubc.ca

**Keywords:** apple, linear discriminant analysis, prediction models, principal component analysis, sensory evaluation, textural evaluations, instrumental evaluations, TA.XT*plus* Texture Analyzer

## Abstract

Textural characteristics of fruit are important for their quality, storability, and consumer acceptance. While texture can be evaluated instrumentally or sensorially, instrumental measurements are preferred if they can be reliably related to human perception. The objectives of this research were to validate instrumental measurements with sensory determinations, develop a classification scheme to group apples by their textural characteristics, and create models to predict sensory attributes from instrumental and compositional analyses. The textural characteristics (crispness, hardness, juiciness, and skin toughness) of 12 apple cultivars were evaluated on new and established cultivars. Fruit was also evaluated using five instrumental measurements from TA.XT*plus* Texture Analyzer, and three compositional determinations. The experiment was repeated for analysis and validation purposes. Principal component (PC) analysis revealed that 95.88% of the variation in the instrumental determinations could be explained by two components (PC 1 and PC 2); which were highly correlated with flesh firmness and skin strength, respectively. Four textural groups of apples were identified, and the accuracy of classification was established at 94.44% by using linear discriminant analysis. The predictive models that were developed between the sensory and instrumental-compositional data explained more than 85% of the variation in the data for hardness and crispness, while models for juiciness and skin toughness were more complex. The work should assist industry personnel to reduce time-consuming and costly sensory testing, yet have an appreciation of the textural traits as perceived by the consumer.

## 1. Introduction

Textural attributes of apples affect their quality and storability and also customers’ acceptance of the fruit [1,2,3]. Sensory evaluations have been utilized in the selection of new fruit cultivars and evaluation of the impact of different storage and handling practices on the textual characteristics of apples [4,5]. However, conducting sensory panels to evaluate the textural attributes of fruits is not always feasible or affordable. Moreover, the sheer volume of sensory tests required by breeding programs, postharvest laboratories, and industry settings, may require hundreds of evaluations per setting; this necessitates application of the more cost- and time-effective methods, such as instrumental measurements in quality assessments. However, the data obtained from the instruments must be validated using sensory tests before these measurements can be reliably adopted for mainstream application or quality control.

Apples are one of the most produced and consumed fruits in the world. Apple cultivars have a broad range of textural characteristics that could vary by their genetics and environmental factors (e.g., production practices, handling methods, transportation, and storage conditions) [6]. There has been an ongoing attempt from the scientific community to develop predictive sensory textural models for apples using a variety of invasive and non-invasive instrumental measurements to eliminate the need for conducting repetitive sensory tests in different settings, especially in the breeding programs [7,8,9,10,11,12,13,14]. Validating instrumental measurements with sensory determinations is an important step in the use of mechanical assessments for textural evaluations. Once validated, instrumental measurements provide powerful selection and decision-making tools for researchers and industry experts.

Simple apple flesh firmness puncture tests can be conducted using different instruments. For example, penetrometers (i.e., Effegi) could be mounted on a drill press in older versions [15] or performed by more sophisticated instruments (i.e., MTS traction machine [9] and Mohr Digi-Test-2 [16,17]) that allow researchers to have a continuous record of the force with penetration [17]. Care must be taken when comparing determinations taken from the different fruit-firmness instruments, because they generate a unique set of data and sample preparation may not be same. This necessitates the need for development of a separate set of predictive models for each instrument.

The size and shape of apple samples and whether the sample is with (unpeeled) or without (peeled) skin play important roles in the type of data collected from the quality assessments conducted using different instrumental measurements. In the majority of the predictive sensory studies, researchers have used peeled apples or small flesh samples [11,12] in their studies, considering the instructions of the instruments and their sample preparation protocols. Grotte et al. [18] penetrated peeled and unpeeled apples and reported that the skin contributed 57–61% to the overall firmness of an apple. Costa [19] also reported that fruit peel contributed about 60% to penetrometer firmness measurements (with considerable differences among cultivars). Their results indicate the importance of the type of sample in studying the instrumental measurements.

Unpeeled samples were utilized in some instrumental measurement studies of apple texture [9,18]. The TA.XT*plus* Texture Analyzer (Stable Micro Systems Ltd., Godalming, Surrey, UK) is a relatively new universal textural instrument that is gaining ground in research and industry settings. It can conduct penetration tests, on unpeeled or peeled apple samples, in order to quantify the textural characteristics of fruit. This instrument can be equipped for semi-automated analyses, thereby providing a substantial savings to industry.

Corollaro et al. [11] and Costa et al. [20] investigated the relationships among the sensory attributes and mechanical and acoustic instrumental measurements obtained from a TA.XT*plus* Texture Analyzer (here thereafter referred as TA.XT*plus*) using large apple collections and developed predictive sensory models using partial least square (PLS) analysis. In both studies, researchers used small samples dissected from apple flesh. This excluded the possibility of understanding the overall textural quality of the apples, which is dependent on the properties of apple flesh and the contribution of apple skin. In addition, the developed predictive models should be easily adopted and applied by experts from different sectors of the apple supply chain [11].

Considering the limitations of the available literature, the main objectives of this research were the following: (1) validate the selected instrumental textural parameters obtained from the TA.XT*plus* with sensory determinations, using principal component analysis; (2) develop an apple texture classification scheme and test its accuracy of classification, using linear discrimination analysis; and (3) develop easily-applicable linear and nonlinear regression models, for predicting the sensory attributes of apples from instrumental determinations obtained from the TA.XT*plus* (i.e., accounting for the skin strength contribution in the firmness perception) and basic compositional data.

## 2. Materials and Methods

### 2.1. Apple Cultivars

Twelve apple cultivars, four from each of three harvest periods (early-, mid-, late-harvest), were included in the study to provide a broad range of textural sensory attributes. Nine of the selected cultivars were common apple cultivars, readily available in the market.

Three cultivars (McIntosh, Imperial Gala, and Silken) were from the early-harvest period; three cultivars (Honeycrisp, Ambrosia, and Aurora Golden Gala) were from the mid-harvest period, and three cultivars (Nicola, Common Red Delicious, and Fuji) were from the late-harvest period. An additional three cultivars were selected from new unnamed selections (SuRDC1, SuRDC2, and SuRDC3), from the Summerland Research and Development Centre in Canada (SuRDC) breeding program (Summerland, BC, Canada). One new cultivar was selected for each of the harvest periods, based on their superior textural characteristics. This broaden the range of the studied textural attributes and allowed the models to be appropriate for both established and up-and-coming (new) cultivars.

The fruit in this research were obtained from an experimental orchard at SuRDC, not a retail outlet, with the exception of Honeycrisp which was purchased from a nearby commercial orchard. None of the fruit were waxed or packaged using commercial packinghouse practices. This meant that the cultivars were sorted to exclude fruit that was unrepresentative in condition (damaged or defective), size, and color, as typically performed prior to sensory assessments [5,12]. By removing fruit that was damaged or defective, particularly large or small, or uncharacteristically red or green, a ‘uniform’ sample was available for the sensory panel. The apples were stored in air, at 0.5 °C, for 5 to 8 weeks and warmed to room temperature overnight prior to analysis.

### 2.2. Sensory Assessment

Apple sensory tests have been conducted regularly at the SuRDC Sensory Laboratory for many years. Panelists (*n* = 12) for this project were selected based on their interest, availability, and previous experience from staff at SuRDC or Summerland Varieties Corp (Summerland, BC, Canada). The ethics approval for the research was obtained from the Agriculture and Agri-Food Canada Human Research Ethics Committee, and informed consent was obtained for experimentation with human subjects. The panel consisted of three men and nine women ranging in age from 27 to 57 years. Four of the panelists were new to sensory profiling of apples and attended a training session to become familiar with the sensory textural attributes (crispness, hardness, juiciness, and skin toughness), as utilized by Cliff and Bejaei [12].

These attributes, briefly, are defined as follows: (i) crispness, “the pressure build-up and crunching sound that is heard as the tissue breaks or shatters, when biting into the apple”; (ii) hardness, “the resistance to compression when the sample is compressed between the molar teeth”; (iii) juiciness, “the relative juice release when compressing the apple with the back molars”; and (iv) skin toughness, “the relative ease of breakdown of skin in the mouth during chewing with the molar teeth, to prepare the apple for swallowing” [5,14].

Food standards were prepared for each of the textural attributes, which anchored the scales and provided a physical standard for the panelists to use as a reference, as described by Cliff and Bejaei [12].

In the training session, the new panelists practiced scoring apples with different textural attributes. All panelists reviewed the sensory methodology and practiced scoring samples just prior to starting each sensory session.

Assessments were conducted at the SuRDC sensory laboratory in individual booths under red light using Compusense *five^®^* software (Compusense Inc., Guelph, ON, Canada). Apple slices (⅛ apple), from unpeeled fruit, were excised from the sun/shade transition zone. They were assigned a three-digit code and presented in random order on white trays.

The standards for the textural attributes were presented in 30 mL plastic cups and presented on separate trays, simultaneously with the sample trays.

The intensity of the textural attributes was scored on 100 unit unstructured line scales, with low and high marked at 10 and 90 units, respectively. The food standards that were utilized for the anchors were placed at 10 and 90 for crispness, hardness and juiciness, and 50 for skin toughness [14]. The mid-point (50 units) of each scale was marked with a small vertical line.

Considering the expected within cultivar and within fruit variations in natural agricultural products, the experiment was repeated twice, and the datasets were named Experiment #1 and Experiment #2. Panelists evaluated four cultivars from the same harvest period, in triplicate, in each session (in random orders) in Experiment #1, and repeated the assessment in another session (with another random order) in Experiment #2. Panelists were unaware that the cultivars were being included in triplicate, or that the experiment was being repeated (performed twice). The two sessions from the same harvest period were scheduled no more than four days apart. Panelists participated in a total of six sensory sessions, evaluating 12 samples from four cultivars per session. Data from two experiments were compared.

### 2.3. Instrumental Firmness Measurements

Penetrometer measurements were conducted using TA.XT*plus* on 12 apple cultivars (9 apples per cultivar per experiment). Two instrumental test days for each harvest period (totaling 6) were conducted a day before and a day after the related sensory session.

TA.XT*plus* was fitted with an 8 mm cylindrical stainless-steel probe penetrating to a depth of 10 mm at a speed of 10 mm/s and with a load cell of 30 kg in the current study. One measurement per fruit was conducted in the sun–shade transition zone in the equatorial region of the fruit. The skin on the fruit was intact and apples were supported on a circular support ring during measurements. The instrument was operated in compression mode with a trigger force of 0.1 N and force-distance curves were drawn from data collected at a rate of 200 points/s.

**Figure 1:**
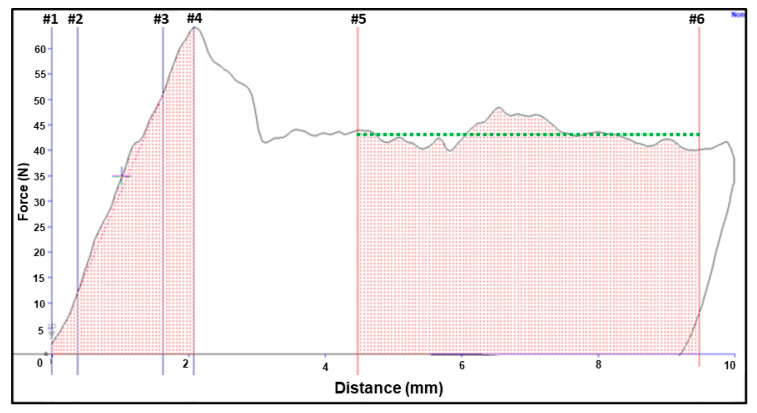
Example of force-distance curve obtained from TA.XT*plus* Texture Analyzer (Stable Micro Systems Ltd., Godalming, Surrey, UK) resulting from penetration of an unpeeled apple. The TA.XT*plus* was equipped with an 8 mm cylindrical stainless-steel probe, and operated to a depth of 10 mm at a speed of 10 mm/s. The parameters used for interpretation of the curves are described in Table 1, using the anchor numbers located along the upper edge of the diagram. The macro used for calculation of the parameters is presented in Table S1.

To select and define parameters for this study, several research papers were considered [9,17,18,21]. Exponent software (Stable Micro Systems Ltd., Godalming, Surrey, UK) was utilized to calculate six parameters from the force-distance curves using a macro, as listed in Table 1 and diagrammatically illustrated in Figure 1. A macro was developed that moved the cursor to specific locations on the curve and performed preprogrammed functions and calculations, as shown in Table S1.

### 2.4. Basic Compositional Measurements

Titratable acidity (*TA*) and soluble solids concentrations (*SSC*) were evaluated in this study, since they are the accepted indices for tracking fruit maturity—an underlying factor in fruit texture. *TA* and *SSC* were measured after the texture measurements from composite juice samples extracted from the top half of three apples (i.e., three samples per cultivar per test date). *TA* was determined using a Model 848 Titrino Plus titrator (Metrohm, Herisau, Switzerland) and reported as g/L malic acid. *SSC* were measured using the Refracto 30PX refractometer (Mettler Toledo, Columbus, OH, USA) and reported in percent (%).

Expressed juice (*EJ*) measurements were conducted, using a modification of the compression method described by Mehinagic et al. [22]. A disc of flesh (21 mm diameter) was excised from the sun/shade transitions zone at the equator of the fruit, using a #14 corkborer. Then, this disc was cut to 13 mm (height) using a 13 mm custom cutting tool (2 utility-knife blade fixed to metal handle), weighed, placed on two layers of Whatman #4 filter paper, and covered with a third piece of filter paper. The disc of flesh was compressed to 80% strain and held for 5 s to express the juice with the TA.XT*plus*. The apple tissue was blotted to remove any excess juice and weighed again. The weight (g) of the *EJ,* removed by compression, was calculated and reported as grams.

### 2.5. Statistical Analysis

Experiment #1 and Experiment #2 datasets from the sensory tests (*n* = 432), instrumental (*n* = 108), *EJ* (*n* = 108) and other basic compositional (*TA*, *SSC*) (*n* = 36) measurements were separately screened to identify any outliers before calculating means for three replicates of each cultivar. Standardized *z*-values were calculated for each variable in both experiments separately, and then any data points with a *z*-value above |3.21| were removed from the datasets. The removed outliers were less than 4.6% of the data presented in a variable. The performance of one judge in the hardness sensory variables and one judge in the skin toughness sensory evaluations in both experiments were determined to be unacceptable. Since the collected data were too low, without any variation among cultivars, these data were removed from both datasets.

After the removal of outliers, means were calculated for three replicates of each cultivar for all variables in both experiments (12 cultivars × 3 replications = 36 sample size per variable in each experiment). A dataset consisting of 72 rows (36 data cells from each experiment) was developed by including three replicates for each apple cultivar in each experiment. Then, independent *t*-test statistics were utilized to compare similar variables in Experiment #1 and #2 with each other. Pearson correlation coefficients were also calculated to study the linear bivariate relationships between sensory, instrumental, and compositional variables with each other using JMP software (JMP^®^, Version 15.0.0, SAS Institute Inc., Cary, NC, USA).

Principle component analysis (PCA) was conducted using the standardized mean scores, for the units of measurement that were different for the five instrumental parameters as measured by the TA.XT*plus* (i.e., *Fs, Ws, Grad, D,* and *Ff*). then, the PCA was supplemented with additional variables (sensory mean scores) using a PCA option in JMP software to explore their relationships with the developed PCs [13]. This option correlated the mean sensory scores with the PC loadings and allowed the sensory attributes to be positioned in the biplot.

The sensory attributes crispness, hardness, and skin toughness were considered for classification of the apple cultivars into different textural categories. Then, linear discriminant analysis (LDA) (sometimes referred to as canonical discriminant analysis) was applied to evaluate the accuracy of the developed textural categories using five TA.XT*plus* measurements [23,24]. The first canonical discriminant functions (which are linear combinations of original variables) were calculated to characterize each sample within the multidimensional space. Then, the canonical discriminant functions were developed, and finally the cases were assigned to the groups based on the highest probability calculated for the groups. Linear combinations of variables (i.e., canonical variables) could be detected using LDA by maximizing the between-class variations and minimizing the within-class variations.

Predictive sensory models were developed using the instrumental and compositional measurements as potential predictor variables and sensory textural measurements as dependent variables. Multiple linear and nonlinear regression models were selected depending on the nature of the relationship between the selected predictor variables and the sensory variables. The prediction powers (*R*^2^) and root mean square errors (RMSE) were considered in the selection of the best-fit prediction models. Predictive models were developed twice using data from each experiment independently. Each model was tested using the data obtained in the other experiment, for validation of the model. then, the final models were calculated using the average coefficients from the two models, and finally the models were tested on a pooled dataset from the two experiments. All statistical tests were conducted using JMP software, at α = 0.05 significance level.

## 3. Results and Discussion

### 3.1. Comparisons of the Results of Two Experiments

Results of the independent *t*-test analyses (Table 2) indicated that there were no significant differences between Experiment #1 and Experiment #2 for the studied variables, and judges were capable of reproducibly evaluating the sensory attributes. Moreover, the broad ranges reported for the variables (i.e., minimum, maximum) indicated the applicability of the results for a diverse collection of apple cultivars that have sensory, instrumental, and compositional characteristics within the studied ranges (Table 2).

### 3.2. Bivariate Pearson Correlation Coefficients

Considering the lack of significant differences between the two experiments in the studied variables, the bivariate linear correlation coefficients (*r*) among the instrumental (*Fs*, *Ws*, *Grad*, *D*, and *Ff*), basic compositional (*EJ, TA*, and *SSC*) and sensory (hardness, crispness, juiciness, and skin toughness) variables were investigated and reported in Table S2 using the pooled dataset. These results were also considered in the selection of the variables for the development of the predictive models to avoid multicollinearity problems.

Sensory crispness and hardness evaluations were strongly positively correlated with each other (*r* = 0.93), as well as with sensory juiciness*, Fs, Grad,* and *Ff* (0.53 ≤ *r* ≤ 0.91) (Table S2), whereas they were strongly negatively correlated with *D* and *TA* (−0.60 ≤ *r* ≤ −0.47) (Table S2). Crispness was also positively correlated with *EJ* (*r* = 0.54) and negatively correlated with skin toughness (*r* = −0.27). This indicated that crispier apples were perceived to be juicier with thinner skin. Mehinagic et al. [9] also reported that sensory crispness and chewiness variables were strongly and positively correlated with *Fs* and *Ff* parameters. Hardness was also positively correlated with the *SSC* variable and that was also reported by Cliff and Bejaei [12].

Sensory juiciness was positively correlated with *Grad*, *Ff*, and *EJ*, while being negatively correlated with sensory skin toughness, *D* instrumental measurement, and *TA* values. In the study by Mehinagic et al. [9], *Grad* was also positively correlated with juiciness sensory evaluations. Iwanami et al. [25] also reported that sensory juiciness was positively correlated with instrumental measurements of firmness and water capacity. The juiciness perception is a complex sensation in the mouth [22,25] and is not expected to show a strong linear relationship with instrumental variables [25,26].

Sensory skin toughness was positively correlated with *Fs*, *D*, *Ws*, and *TA* variables but it was negatively correlated with *EJ*. Cliff and Bejaei [12] also reported a similar relationship between skin toughness and *TA* and that was mostly driven by the McIntosh apple which has a tough skin, while being considered to be a tart apple. Skin strength showed a positive correlation with the juiciness in the study by Costa [19], as he included a storage period where apples with thicker skin lost less water.

### 3.3. Principal Component Analysis Using TA.XTplus Measurements and Supplementary Sensory Textural Variables

**Figure 2:**
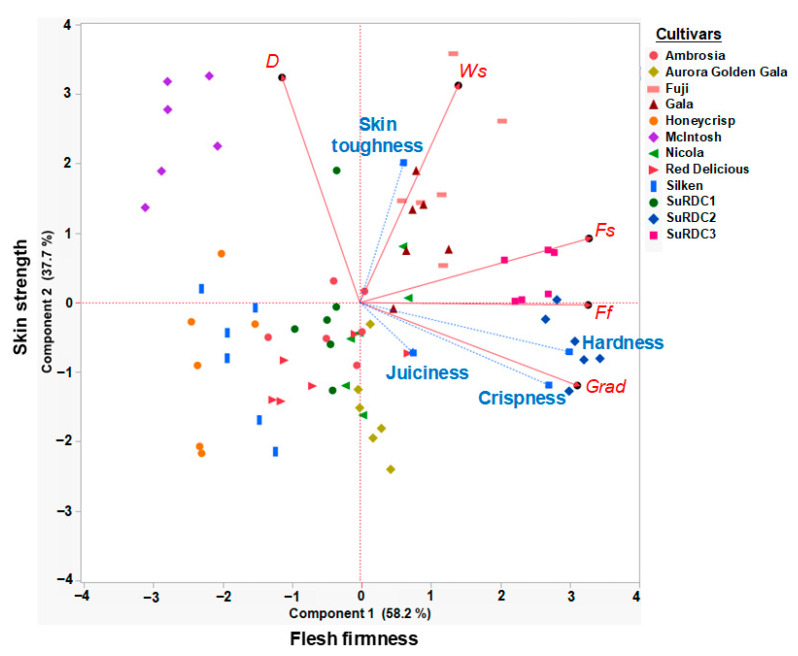
Principal component analysis biplot calculated using standardized mean values of five TA.XT*plus* Texture Analyzer parameters (*Fs*, *Ws*, *Grad*, *D*, and *Ff*). Instrumental measurements, as described in Table 1, are identified by red lines. Apple cultivars are identified with symbols of unique colors and shapes. Sensory textural attributes (crispness, hardness, juiciness, and skin toughness) are identified by blue lines, and positioned on this plot using correlation analysis (see Section 2.5).

The PCA was conducted using TA.XT*plus* measurements from the pooled dataset to validate and interpret the selected measurements before their classification or inclusion in the predictive models. Results of the PCA using five instrumental measurements (*n* = 72 means) indicated that 95.88% variation in the data could be explained using only two principal components (Figure 2), with 58.18% and 37.71% of the variation explained by PC 1 and PC 2, respectively.

The vectors for *Ff*, *Fs*, and *Grad* were heavily associated with PC 1 (with loadings of 0.95, 0.95, and 0.91, respectively), and explained more than half of the variation in the samples. PC 1 was positively and strongly correlated with sensory hardness (*r* = 0.87) and crispness (*r* = 0.79), as evident from the loadings reported for the supplementary variables, and small angles between the sensory and instrumental variables. Therefore, the variables associate with PC 1 were collectively described as “flesh firmness variables” (Figure 2). In contrast, the vectors for *D* and *Ws* were heavily associated with PC 2 (with loadings of 0.94 and 0.90, respectively), and explained slightly more than one-third of the variation in the samples. PC 2 was positively and significantly correlated with skin toughness (*r* = 0.58), as evident from the loadings reported for the supplementary variables and small angle between the sensory and instrumental variables Therefore, the variables associated with PC 2 were collectively described as “skin strength variables” (Figure 2).

However, sensory juiciness was not particularly useful for explaining the variation in the dataset, as evident from low loadings reported between this variable and PC 1 (*r* = 0.22) and PC 2 (*r* = −0.21) and the short length of vector (as compared with the length of other vectors) (Figure 2). Such findings are consistent with other studies that have reported that perceived juiciness is not linearly associated with instrumental variables [22,25]. As a result, the predictive model for sensory juiciness could not be developed exclusively from the Texture Analyzer data, and it was necessary to include compositional measurements.

Mehinagic et al. [9] reported comparable PCA results by analyzing the force-distance curve data obtained from unpeeled apples using an MTS (Syneregie 200H) traction machine. In their study, PC 1 explained 66.4% of the variation among apple samples and correlated with flesh firmness related variables. In their study, PC 2 described 27.4% of the variation, explained by skin strength related variables. The *D* parameter was loaded on PC 2 and it was negatively correlated with firmness measurements in their study. Costa [19] also reported that the skin strength-related variables in his study were negatively correlated with the firmness measurements.

### 3.4. Evaluating the Accuracy of Apply Textural Classifications Using TA.XTplus Measurements

Ballabio et al. [24] divided their samples into two groups based on the crispness scores, and then conducted LDA. In the current study, mean sensory scores for crispness, hardness, and skin toughness evaluations were considered in developing four apple texture classifications (groups). The first group included the samples with the lowest crispness ($\bar{x}$ < 49) and hardness ($\bar{x}$ < 39) scores which had a tough skin. This group was entitled “soft flesh with tough skin”. McIntosh apple was in this category. The second group included apples with low crispness ($\bar{x}$ = 50–59) and hardness ($\bar{x}\;$= 40–49) scores, and thin to moderate skin toughness. This group was called: “soft flesh with thin to moderate skin toughness”. Honeycrisp, Silken, and Red Delicious cultivars were in this category. The third group included apples with moderate mean crispness ($\bar{x}$ = 60–69) and hardness ($\bar{x}=\;$50–64) scores, and moderate skin toughness. This group was entitled: “moderate flesh hardness and skin toughness”, and Ambrosia, Gala, Fuji, SuRDC1, Aurora Golden Gala, and Nicola cultivars were grouped in this category. The last (fourth) group included cultivars with the highest crispness ($\bar{x}$ > 70.0) and hardness ($\bar{x}$ > 65.0) scores but a moderate skin toughness. This group was called: “hard flesh with moderate skin toughness” and included SuRDC2 and SuRDC3 cultivars. After classifying 12 cultivars into four groups, LDA was applied to evaluate the accuracy of the developed textural categories using five selected TA.XT*plus* parameters.

For LDA, the first and the second canonical discriminant functions explained 93.16% and 6.43% of variation in samples, respectively (i.e., 99.59% of cumulative variance). Wilks’ Lambda value was 0.003 (approximate *F*-ratio (15, 177) = 30.61, *p* < 0.0001), and the Entropy *R*^2^ (a measure of fit) was 0.88. Apples in the first and fourth groups were all classified correctly, whereas only two samples from each of the second and third groups were classified incorrectly or misclassified (Table 3). This meant that the classification rate was 94.44% accurate (misclassification rate = 5.56%) and was very successful [23].

**Figure 3:**
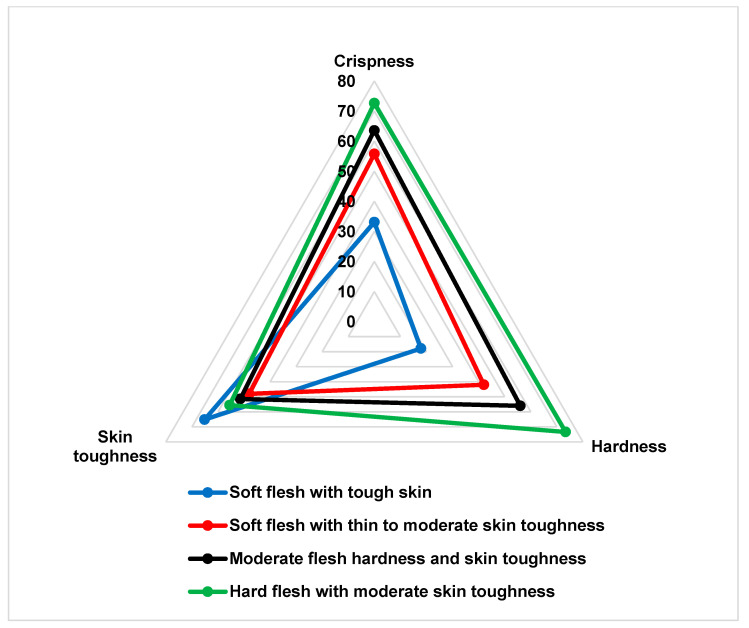
Sensory textural profile showing the mean scores (*n* = 72) for the four apple textural groups, which were identified by PCA and validated by linear discriminant analyses, with the five selected TA.XT*plus* parameters.

Results of the linear discriminant analysis revealed that the developed apple texture groups had unique sensory characteristics (Figure 3). This indicates that the instrumental measurements can be successfully utilized in classifying and predicting the sensory textural profile of apple cultivars.

### 3.5. Predictive Sensory Models

The best-fit models for the prediction of apple crispness, hardness, and skin toughness were all multiple nonlinear regression models, because *Ff* showed a quadratic relationship with these three sensory output variables. Five studied instrumental variables were considered to be the potential predictive variables in the development of models for these three sensory attributes. These output variables were also strongly correlated with the PCA components (either PC 1 or PC 2), as discussed in the PCA results. As a result, the compositional data were not included in the models so that apple supply chain experts can simply use a TA.XT*plus* penetrometer test to predict these three sensory variables without the need for the compositional measurements. However, the apple juiciness perception of panelist could be best predicted by a multiple regression model which required the inclusion of EJ measurements as a predictor variable (as discussed previously). Assumptions of the regression analysis were considered in the development of the models and multicollinearity among the selected individual predictor variables were avoided considering the Pearson correlation coefficients reported in Table S2.

The quadratic effect of *Ff* and linear effect of *D* explained a significant proportion of the variance in the sensory crispness scores in both Experiment #1 (*F*_(3, 31)_ = 74.56, *p* < 0.0001, *R*^2^ = 0.88, *R*^2^_Adjusted_ = 0.87) and Experiment #2 (*F*_(3, 32)_ = 64.66, *p* < 0.0001, *R*^2^ = 0.86, *R*^2^_Adjusted_ = 0.85) datasets. The validations of the Experiment #1 crispness data with the Experiment #2 crispness model and vice versa revealed that 86.47% and 84.31%, respectively, of the variations in each dataset could be explained by the models developed using the other dataset. Calculating average coefficients from the two models resulted in the development of the crispness prediction model presented in Table 4. The developed model could explain 85.76% of variation in the crispness data, from the pooled dataset.

The quadratic effect of *Ff* and linear effect of *D* explained a significant proportion of the variance in the sensory hardness scores in both Experiment #1 (*F*_(3, 31)_ = 77.04, *p* < 0.0001, *R*^2^ = 0.88, *R*^2^_Adjusted_ = 0.87) and Experiment #2 (*F*_(3, 32)_ = 96.76, *p* < 0.0001, *R*^2^ = 0.90, *R*^2^_Adjusted_ = 0.89) datasets. The validations of the Experiment #1 hardness data with the Experiment #2 hardness model and vice versa revealed that 87.30% and 88.75%, respectively, of the variations in each dataset could be explained by the models developed using the other dataset. Calculating average coefficients from the two models resulted in the development of the hardness prediction model presented in Table 4. The developed model could explain 88.82% of variation in the hardness data, from the pooled data set.

The crispness and hardness predictive models explained more than 85% of the variation in the samples. The results indicated that these two sensory attributes can be easily predicted using the TA.XT*plus*. Flesh texture models have been successfully developed using different instrumental firmness measurements in the past [11,12,13,14,21,24,26]. Chang et al. [13] investigated the use of instrumental measurements in evaluating sensory crispness and the changes in sensory crispness during storage of a Honeycrisp apple breeding family. They reported that crispness was more correlated to force (equivalents of D and Fs measured by the mechanical-acoustic test) in their study rather than acoustic measurements. The validation and adjusted *R*^2^s in the current study are very close to the actual *R^2^*s for both crispness and hardness models. This indicates the reliability, precision, accuracy, and repeatability of the developed models in the current study.

The quadratic effect of *Ff* and linear effect of *Ws* explained about half of the variance in the values of sensory skin toughness in both Experiment #1 (*F*_(3, 32)_ = 13.73, *p* < 0.0001, *R^2^* = 0.56, *R^2^*_Adjusted_ = 0.52) and Experiment #2 (*F*_(3, 30)_ = 7.29, *p* = 0.0008, *R^2^* = 0.42, *R^2^*_Adjusted_ = 0.36) datasets. The validations of the Experiment #1 skin toughness data with the Experiment #2 skin toughness model and vice versa revealed that 51.87% and 38.65%, respectively, of the variations in each dataset could be explained by the models developed using the other dataset. Calculating average coefficients from the two models resulted in the development of the skin toughness prediction model presented in Table 4. The developed model could explain 48.51% of variation in the hardness data, from the pooled data set.

The skin toughness perception (tender or tough) can be influenced not only by the skin strength but also by the flesh firmness and apple juiciness. Grotte et al. [18] studied the contribution of the apple peel to the overall firmness of apple in four apple cultivars and indicated that there was no difference among the studied cultivars in the influence of skin on the fruit firmness. However, Costa [19] studied the contributions of the skin on the fruit firmness in 65 cultivars and reported considerable differences among the cultivars in the role of skin on the fruit firmness. The physical thickness of the epidermis of apples does not necessarily reflect the perceived toughness of the skin. In the current study, considerable variations were detected among the cultivars in their skin strengths which were observable in the second component of the preference mapping analysis.

The linear effects of *Grad*, *Ws*, *Ff* and *EJ* explained half of the variance in the values of sensory juiciness in both Experiment #1 (*F*_(4, 30)_ = 8.97, *p* < 0.0001, *R*^2^ = 0.54, *R*^2^_Adjusted_ = 0.48) and Experiment #2 (*F*_(4, 31)_ = 8.28, *p* < 0.0001, *R^2^* = 0.52, *R^2^*_Adjusted_ = 0.45) datasets. The validations of the Experiment #1 juiciness data with the Experiment #2 juiciness model and vice versa revealed that 50.26% and 46.53%, respectively, of the variations in each dataset could be explained by the models developed using the other dataset. Calculating average coefficients from the two models resulted in the development of the juiciness prediction model presented in Table 4. The developed model could explain 50.99% of variation in the juiciness data, from the pooled dataset.

Sensory juiciness is considered to be a complex variable because several physicochemical of the fruits (i.e., proportion of air spaces to its total tissue volume, cell dimensions, cell wall thickness, and water distribution in intercellular, intracellular, and within the cell wall) can influence the perception of sensory juiciness [22,25,27]. Inclusion of the *EJ* parameter in the juiciness model was necessary, as discussed previously, because different factors affect the juiciness perception of humans during mastication of fruits and not all can be captured by fruit texture instrumental measurements. Iwanami et al. [25] also conducted instrumental juice measurements using two different methods (i.e., by centrifuging small samples twice or drying other samples) and considered the instrumental juice measurements in the development of predictive models for sensory juiciness. Cliff and Bejaei [12] also included absorbed juice compositional measurement in a juiciness prediction model in addition to using other instrumental measurements.

The juiciness model might be improved in the future by incorporating an alternate juice measurement that might correlate better with perceived juiciness [20,22,25], or incorporate an additional compositional variable, such as dry matter [28].

## 4. Conclusions

Results of this study indicated that five instrumental textural determinations (*Fs*, *Ws*, *Grad*, *D*, and *Fs*) from the TA.XT*plus* correlated well, and could be validated, with sensory textural evaluations. These five instrumental parameters can successfully evaluate apple flesh firmness and skin strength, as well as classify the apples into four textural categories, with a high accuracy rate (94.4%).

The strength of this research lies, in part, with the use of the universal Texture Analyzer (TA.XT*plus*). It was capable of providing determinations on unpeeled whole apples. These determinations, along with the predictive models, allow the sensory textural characteristics of apples (crispiness, hardness, and skin toughness) to be estimated without the need for additional compositional or sensory data. Future research exploring alternate instrumental measurements or novel variables might be able to improve the prediction power of the juiciness model.

This work would be useful for apple breeding programs and packinghouses that require high-volume textural analyses on a daily basis. The application of the validated models would reduce or eliminate the need for costly and time-consuming sensory testing. If the TA.XT*plus* were equipped for semi-automated determinations, it would provide additional convenience and savings to industry.

The models in this research were developed using a particularly unique collection of apple cultivars that included experimental cultivars with superior textural traits. This meant that the models are appropriate for existing cultivars, and new cultivars that are not yet in the marketplace. Furthermore, the models were developed using unpeeled apples, which reflect more closely the complex events that occur in the mouth when a consumer bites into a whole apple.

## Figures and Tables

**Table 1:** List of instrumental parameters, abbreviations, and units of measurement associated with the instrumental measurements on the TA.XT*plus* Texture Analyzer.

Parameter Abbreviation	Units of Measure	Description of Parameter	Method of Calculation and Location on Force-Distance Curve
*Fs*	Newton (N)	The maximum force (recorded on y-axis) required to rupture apple skin and flesh	Shown by anchor #4 on Figure 1
*Ws*	Nmm	Mechanical work conducted to rupture skin and flesh	Calculated from the triangle area under the curve between anchors #1 and #4 on Figure 1
*Grad*	N/mm	The gradient on the force-distance curve between 20% and 80% ^a^ of *Fs* to measure the slope of the firmness	Calculated from the slope between anchors #2 and #3 on Figure 1
*D*	mm	The probe position (on x-axis) at *Fs*	Measured from the distance between anchors #1 and #4 on the x-axis of Figure 1
*Ff*	N	The average force required to puncture the flesh between 4.5 mm and 9.5 mm ^a^	Calculated by the average of force recorded between anchor #5 to #6 on Figure 1
*Wf*	Nmm	Work to rupture the flesh between 4.5 and 9.5 mm ^b^	Calculated from the marked rectangle area under the curve between anchors #5 and #6 on Figure 1

^a^ Definitions for *Grad* and *Ff* variables were selected by observing 216 curves from 12 apple cultivars to minimize the impact of within cultivar and among cultivar variations. ^b^ Considering a fixed distance between anchors #5 and #6 (i.e., 5 mm), *Wf* was equal to *Ff* multiplied by 5. As a result, it was dropped from further analysis.

**Table 2:** Means, standard errors (*SE*), minimum (Min.), maximum (Max.) and *t*-test results of the comparison of sensory, instrumental, and compositional data obtained in Experiment #1 and Experiment #2 (*n* = 72, *df* = 70).

		Experiment 1				Experiment 2					
		Mean	*SE*	Min.	Max.	Mean	*SE*	Min.	Max.	*t*-Test	Significance (2-Tailed)
Sensory ^a^	Crispness	59.48	1.78	31.50	74.58	61.31	1.78	28.63	74.50	0.72	0.47
	Hardness	51.17	2.55	16.68	75.23	52.64	2.55	16.64	76.82	0.41	0.69
	Juiciness	58.32	0.87	49.00	66.38	59.74	0.87	47.05	68.71	1.16	0.25
	Skin toughness	51.96	1.33	34.59	66.68	52.6	1.33	38.41	66.59	0.34	0.73
Instrumental ^b^	*Fs*	65.22	1.85	48.45	88.20	65.93	1.85	45.84	86.78	0.27	0.79
	*Ws*	87.02	3.24	56.42	116.02	89.86	3.24	50.04	143.39	0.62	0.54
	*Grad*	27.85	0.99	16.19	42.35	27.56	0.99	16.90	40.30	−0.21	0.84
	*D*	2.66	0.06	2.00	3.73	2.69	0.06	2.22	3.58	0.27	0.79
	*Ff*	39.23	1.28	24.83	56.24	39.97	1.28	25.12	55.29	0.41	0.68
Compositional ^c^	Expressed juiciness (*EJ*)	1.83	0.04	1.16	2.23	1.83	0.04	1.13	2.28	0.02	0.98
	Titratable acidity (*TA*)	4.38	0.22	2.66	7.49	4.45	0.22	2.61	9.07	0.21	0.83
	Soluble solids concentrations (*SSC*)	14.94	0.19	11.60	17.00	14.95	0.19	11.60	17.60	0.03	0.98

^a^ Sensory attributes (crispness, hardness, juiciness, and skin toughness) maximum scores are 100 units. ^b^ Determined using the TA.XT*plus* Texture Analyzer (Stable Micro Systems Ltd., Godalming, Surrey, UK), as described in Table 1. ^c^ *EJ*, *TA*, and *SSC* were determined in units of mg/L malic acid equivalent, percent and grams, respectively.

**Table 3:** Classification accuracy matrix for four apple textural groups based on the linear discriminant analysis using five selected TA.XT*plus* parameters (*n* = 72).

Apple textural Group and Name		Predicted Group			
		Group 1	Group 2	Group 3	Group 4
Actual Group	Group 1. “Soft flesh with tough skin”	6	0	0	0
	Group 2. “Soft flesh with thin to moderate skin toughness”	0	16	2	0
	Group 3. “Moderate flesh hardness and skin toughness”	0	2	34	0
	Group 4. “Hard flesh with moderate skin toughness”	0	0	0	12

**Table 4:** Best-fit regression models for predicting sensory attributes from determination with the TA.XT*plus* Texture Analyzer. Models were developed using mean values (*n* = 72).

Sensory Attribute	Best-fit Regression Model ^a^	*F*-Value ^b^	Prediction Power *R*^2^ (%)	Adjusted R^2^ (%)	Root Mean Square Error (RMSE)
**Crispness**	= (−12.36) + (3.85 × *Ff*) + (−0.03 × *Ff*^2^) + (−8.66 × *D*)	134.49	85.76	85.12	4.14
**Hardness**	= (−45.18) + (4.12 × *Ff*) + (−0.03 × *Ff*^2^) + (−6.79 × *D*)	177.57	88.83	88.33	5.23
**Skin Toughness**	= (83.44) + (−2.56 × *Ff*) + (0.03 × *Ff*^2^) + (0.24 × *Ws*)	20.73	48.51	46.17	5.54
**Juiciness**	= (42.42) + (−0.26 × *Grad*) + (−0.09 × *Ws*) + (0.42 × *Ff*) + (8.04 × *EJ*)	17.17	50.99	48.02	3.30

^a^ Variables in regression models are instrumental parameters determined using the TA.XT*plus* Texture Analyzer (Stable Micro Systems Ltd., Godalming, Surrey, UK) as described in Table 1. ^b^ Significance of *F*-values < 0.0001.

## Data Availability

Not applicable.

## Supplementary Materials

The following is available online at https://www.mdpi.com/2304-8158/10/2/384/s1, Table S1: Macro for calculating six parameters on the force-distance curves, developed for the TA.XT*plus* Texture Analyzer using Exponent software, Table S2: Pearson correlation coefficients (***r***) and *p*-values calculated between mean sensory, instrumental, and compositional determinations (*n* = 72).
