# Supplementary material for: Modelling and Classification of Apple Textural Attributes Using Sensory, Instrumental and Compositional Analyses
Source: Foods. 2021 Feb 10;10(2):384. doi: 10.3390/foods10020384 (PMC7916372; doi:10.3390/foods10020384)
Supplement: Supplementary file 1 [file foods-10-00384-s001.pdf]

foods MDPI

Supplementary Material

Table S1. Macro for calculating six parameters on the force-distance curves, developed for the TA.XT*plus* Texture Analyzer using Exponent software (Stable Micro Systems Ltd., Godalming, Surrey, UK)

	Program	Flags
1:	Clear Graph Results	
2:	Redraw	
3:	Search Forwards	
4:	Go to Min. Time	
5:	Drop Anchor	
6:	Go to 20 Percent of Max. Force	
7:	Drop Anchor	
8:	Go to 80 Percent of Max. Force	
9:	Drop Anchor	
10:	Gradient (Active vs Active) as Grad (20-80%)	(R)
11:	Go to Absolute +ve Value Force Current Units	
12:	Drop Anchor	
13:	Mark Value (Force (N)) as Fs	(R)
14:	Mark Value (Distance (mm)) as D	(R)
15:	Select Anchor 1, Anchor Not Activated	(F!)
16:	Area (Active vs Active) as Ws	(R)
17:	Go to Distance 4.5 mm	
18:	Drop Anchor	
19:	Go to Distance 9.5 mm	
20:	Drop Anchor	
21:	Mean (Active) as Ff	(R)
22:	Area (Active vs Active) as Wf	(R)

Table S2. Pearson correlation coefficients (*r*) and *p*-values calculated between mean sensory, instrumental [a] and compositional determinations (*n* = 72).

		**Crisp-ness**	**Hardness**	**Juiciness**	**Skin toughness**	***Fs***	***Ws***	***Grad***	***D***	***Ff***	***EJ***	***TA***
Hardness	***r***	**0.93**										
	p-value	<0.0001										
Juiciness	***r***	**0.61**	**0.47**									
	p-value	<0.0001	<0.0001									
Skin toughness	***r***	**−0.19**	**−0.01**	**−0.31**								
	p-value	0.12	0.93	0.01								
Fs	***r***	**0.63**	**0.77**	**0.19**	**0.33**							
	p-value	<0.0001	<0.0001	0.12	<0.01							
Ws	***r***	**0.00**	**0.16**	**−0.14**	**0.63**	**0.64**						
	p-value	0.97	0.18	0.25	<0.0001	<0.0001						
Grad	***r***	**0.75**	**0.80**	**0.25**	**−0.10**	**0.78**	**0.07**					
	p-value	<0.0001	<0.0001	0.03	0.41	<0.0001	0.56					
D	***r***	**−0.60**	**−0.48**	**−0.39**	**0.53**	**−0.07**	**0.70**	**−0.61**				
	p-value	<0.0001	<0.0001	<0.001	<0.0001	0.58	<0.0001	<0.0001				
Ff	***r***	**0.83**	**0.91**	**0.40**	**0.12**	**0.88**	**0.36**	**0.80**	**−0.31**			
	p-value	<0.0001	<0.0001	<0.001	0.34	<0.0001	<0.01	<0.0001	0.01			
Expressed juiciness	***r***	**0.54**	**0.42**	**0.61**	**−0.26**	**0.13**	**−0.18**	**0.22**	**−0.41**	**0.29**		
(*EJ*)	*p*-value	<0.0001	<0.001	<0.0001	0.03	0.29	0.13	0.06	<0.001	0.01		
Titratable acidity	***r***	**−0.57**	**−0.47**	**−0.34**	**0.41**	**−0.11**	**0.36**	**−0.40**	**0.65**	**−0.28**	**−0.53**	
(*TA*)	*p*-value	<0.0001	<0.0001	<0.01	<0.001	0.37	<0.01	<0.001	<0.0001	0.02	<0.0001	
Soluble solids con-	***r***	**0.20**	**0.24**	**0.08**	**0.01**	**0.38**	**0.33**	**0.23**	**0.08**	**0.32**	**−0.23**	**−0.01**
centrations (*SSC*)	*p*-value	0.10	0.05	0.49	0.91	0.001	<0.01	0.05	0.52	0.01	0.05	0.97

[a] determined using the TA.XT*plus* Texture Analyzer (Stable Micro Systems Ltd., Godalming, Surrey, UK) as described in Table 1.
